# Resident attitudes and benefits of mock oral board examinations in radiation oncology

**DOI:** 10.1186/s12909-020-02106-4

**Published:** 2020-06-26

**Authors:** Gabrielle W. Peters, Roy H. Decker, Henry S. Park, James B. Yu, Suzanne B. Evans

**Affiliations:** Department of Therapeutic Radiology, Yale School of Medicine, 35 Park Street, New Haven, CT 06516 USA

## Abstract

**Background:**

Presently, educational programming is not standardized across radiation oncology (RO) training programs. Specifically, there are limited materials through national organizations or structured practice exams for residents preparing for the American Board of Radiology (ABR) oral board examination. We present our 2019 experience implementing a formalized program of early mock oral board examinations (MOBE) for residents in post-graduate years (PGY) 3–5.

**Methods:**

A mixed-methods survey regarding MOBE perception and self-reported comfort across five clinical domains were administered to PGY2–5 residents. MOBEs and a post-intervention survey were implemented for the PGY3–5. The pre and post-intervention score across clinical domains were compared using t-tests. Faculty and residents were asked for post-intervention comments.

**Results:**

A total of 14 PGY2–5 residents completed the pre-intervention survey; 9 residents participated in the MOBE (5/14 residents were PGY2s) and post-intervention survey. This was the first mock oral radiation oncology examination experience for 65% of residents. 100% of residents felt the MOBE increased their clinical knowledge and comfort with clinical reasoning. Overall, there was a trend towards improved resident confidence giving planning dose parameters and (*p* = 0.08). There was also unanimous request for more MOBE experiences from residents and faculty, but time was identified as a significant barrier.

**Conclusions:**

Future directions for this MOBE program are inclusion of more disease sites, better emulation of the exam, the creation of a more rigorous consolidated format testing all sites at once, and consideration for grading of these sessions for future correlation with certifying oral board examination (OBE) performance.

## Background

At present, clinical resident educational programming in radiation oncology (RO) is not standardized across training programs. The American Board of Radiology (ABR) administers three written examinations prior to full certification: physics, radiation biology, and clinical board exams. For these three exams, the American Society of Radiation Oncology (ASTRO) historically has published study guides to aide in preparation for these exams, and Medical Physics Publishing produces practice exams for the physics boards.

In contrast, the ABR oral board exam is administered at the conclusion of one’s first year in clinical practice, and it is the final step in becoming a board certified radiation oncologist. Passing the oral board exam (OBE) requires not only clinical expertise, but also confidence and poise to fluently explain complicated rationale and techniques in a high stress situation. To our knowledge, there are no publicly available OBE grading guidelines, however the ABR does provide a broad informational guideline [1] outlining the breadth of expertise required, including anatomy and clinical evaluation, selection of appropriate therapeutic intervention (including surgery, systemic therapy, and radiation therapy), radiation planning techniques, results of treatment (both oncologic outcomes and toxicity/complication management), as well as significant results of clinical trials.

Although individual resident factors are important in first-time OBE pass rates, training program factors, including the use of mock oral board examinations (MOBE), are also important across graduate medical education (GME) [2, 3]. In other specialties, such as surgery [4, 5], anesthesia [6], and radiology [7] MOBE have been associated with improved preparedness (mentally and emotionally) and improved first-time OBE pass rates. Although MOBE are prevalent in radiation oncology training programs [8], there have been no studies regarding the perceived utility of MOBE for radiation oncology residents and faculty. MOBEs at this institution have historically been optional and predominantly utilized by the chief residents.

The primary aim of this quality improvement project was to see if mandatory and early introduction of MOBEs in a RO department among residents in post-graduate year (PGY) three through five would result in the perception of utility by residents and attendings. This initiative also will measure the impact on resident comfort with the themes tested in OBE, and identify barriers to future expansion.

## Methods

### Mock Oral program

The Yale institutional Human Investigations Committee granted an exemption for this project. This program was designed to complement the current clinical education program to be implemented in the spring of 2018–2019 academic year. The academic year is divided into 6 rotation blocks during which time each resident is paired with an attending and rotates on a disease-specific service. At the time of MOBE inception, PGY2 residents had only completed two rotations and were still becoming acclimated to the clinic. Therefore, it was decided a priori to include PGY3–5 residents in the MOBE implementation. Of the 14 faculty members interacting daily with residents, 10 were available to participate in the MOBE. Participating residents chose two disease sites from the six disease-specific rotations they had been exposed to in the past year, and were then assigned a faculty member and date for their requested disease site. The MOBE date was selected in a manner which would not conflict with clinical expectations or scheduled lectures. Residents prepared for these sessions at their own discretion, although time was limited for preparation. Faculty members were advised to lead 30 min of mock oral examination with each resident, similar to what is experienced during the ABR OBE. There was not formalized faculty training for OBE administration. However, three participating faculty were current or former ABR examiners, and all of the remaining faculty had previously given informal MOBE to the institution’s graduated residents, making them familiar with the format.

The faculty assessed the aforementioned five clinical skills themes commonly tested during OBE. This was followed by 30 min of debriefing and one-on-one feedback. Both sessions were scheduled during 1 week for each resident. All but one participating resident completed both MOBEs. The participating residents (PGY3–5) then completed a post-intervention mixed methods survey (previous Likert scale questions and free-form text for comments and critiques).

### Survey

Anonymous pre and post-intervention mixed methods surveys were developed and distributed to all PGY2–5 residents, in order understand attitudes of all residents towards formalized MOBE. To our knowledge, there are no validated survey tools available to assess RO trainee clinical skill knowledge or comfort. Therefore, the ABR informational guide [1] was used as a guide for author adaptation into 5 clinical skill themes: conducting an appropriate workup, giving evidence based treatment recommendations (including selection of appropriate treatment modality, sequencing, and on-treatment management), acknowledging competing risks and benefits of radiation, evaluation of treatment plans, and demonstrating knowledge of target volume and organ at risk dose constraints and goals. This survey included Likert scale questions (whole integer scale 1–5; very poor/uncomfortable to excellent/very comfortable) as well as free-text qualitative questions regarding utility and barriers to MOBE (Supplemental). Study data were collected and managed using REDCap electronic data capture tools hosted at Yale University [9, 10]. As this program was a pilot, the trainees also served to pilot this survey.

The survey data was analyzed qualitatively and quantitatively. Qualitative assessment included review of free-text comments, identifying themes and reporting the consensus and outlying opinion, if present. Quantitative analysis included t-test analysis of PGY3-5 pre and post-MOBE clinical skill comfort scores. ANOVA tests compared comfort across resident classes. Statistical significance was defined as *p* <0.05. All comparisons between the pre and post intervention survey excluded PGY2 residents, who were not included in the post intervention survey.

## Results

**Fig. 1:**
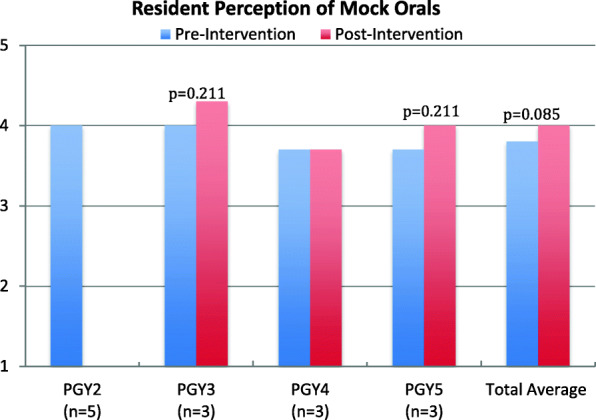
Resident perception of mock oral board examinations. [Likert scale (1-5; harmful to very beneficial)]

There was a 100% response rate of the pre-intervention tool (14/14) and post-intervention tool (9/9) (Table 1) [Survey available in Supplemental Data]. Only 35% of residents (5/14) had experience with a formal case-based oral examination in radiation oncology prior to this study. Resident perception of MOBE utility was increased in the post-intervention survey, with a trend toward significance (*p* = 0.085) [Fig. 1]. Pre-intervention qualitative responses described the anticipated benefits of Socratic teaching as “[it] enables residents to assess their knowledge and evaluate the gaps in training,” “forces [us] to think on the spot,” “information gained through Socratic teaching stays with you longer than that of passive learning,” and “allows synthesis of [fragmented] data you have learned into a useful whole.”

**Table 1:** Survey Cohort

	Pre-Intervention Cohort	Post-Intervention Cohort
PGY2	5	–
PGY3	3	3
PGY4	3	3
PGY5	3	3
Prior experience^a^	5	–

^a^Prior experience with case-based or mock oral training in radiation oncology setting

**Fig. 2:**
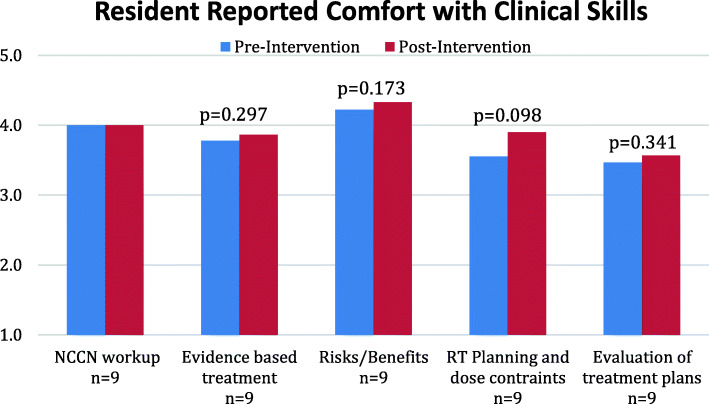
Results of PGY3–5 resident cohort self-reported, average comfort with clinical skills before and after mock oral board intervention. [Likert scale (1-5; very uncomfortable- very comfortable)]. NCCN= National Comprehensive Cancer Network; RT= radiation therapy; PGY= post-graduate year

**Fig. 3:**
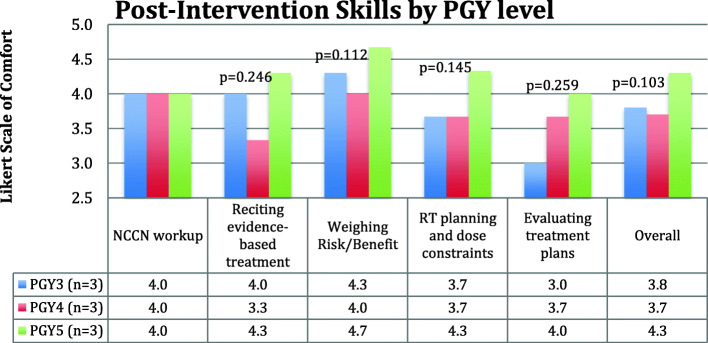
Results of PGY3–5 resident cohort self-reported comfort with clinical skills, broken down by class, after mock oral board intervention

Following MOBE experience, there were numerical improvements in resident comfort in four of the five clinical skill themes level, with a trend toward significance in the area of giving planning dose parameters/constraints (*p* = 0.098) [Fig. 2]. When analyzed by class, PGY3 residents showed a trend to increased comfort with giving guidance on planning and dose constraints (comfortable to very comfortable; *p* = 0.09) while PGY5 residents experienced increased comfort with delivering evidence-based treatment recommendations (comfortable to very comfortable; *p* = 0.09) [Table 2]. Overall, PGY5s appeared more comfortable with the clinical skills themes (Fig. 3), however this did not reach statistical significance. 3/5 residents with prior MOBE experience were in the PGY2 class, meaning that only 2 of the residents completing MOBE had prior experience. We did not perform an analysis comparing these 2 residents to the other 7 because of the small number involved. 100% of residents felt the MOBE increased their clinical knowledge and comfort with clinical reasoning [Fig. 2] and there was unanimous request for more MOBE experiences.

**Table 2:** Comparison of resident-reported comfort with clinical skill themes pre and post-MOBE intervention

	NCCN Workup	Reciting Evidence-based Treatment	Weighing Risk/Benefit	RT Planning and Dose Constraints	Evaluating Treatment Plans
PGY2 (*n* = 5)	2.6	2.4	2.6	2	2.2
PGY3 (*n* = 3)					***p*** **= 0.371**
*p*-value				***p*** **= 0.092**	
Pre-MOBE	4.0	4.0	4.3	3.0	2.7
Post-MOBE	4.0	4.0	4.3	3.7	3.0
PGY4 (*n* = 3)					
*p*-value		***p*** **= 0.211**			
Pre-MOBE	4.0	3.7	4.0	3.7	3.7
Post-MOBE	4.0	3.3	4.0	3.7	3.7
PGY5 (*n* = 3)					
*p*-value		***p*** **= 0.092**	***p*** **= 0.211**	***p*** **= 0.211**	
Pre-MOBE	4.0	3.7	4.3	4.0	4.0
Post-MOBE	4.0	4.3	4.7	4.3	4.0

[Likert scale (1–5, very uncomfortable- very comfortable)]. MOBE = mock oral board examination; *NCCN* National Comprehensive Cancer Network; *RT* radiation therapy; *PGY* post-graduate year

**Fig. 4:**
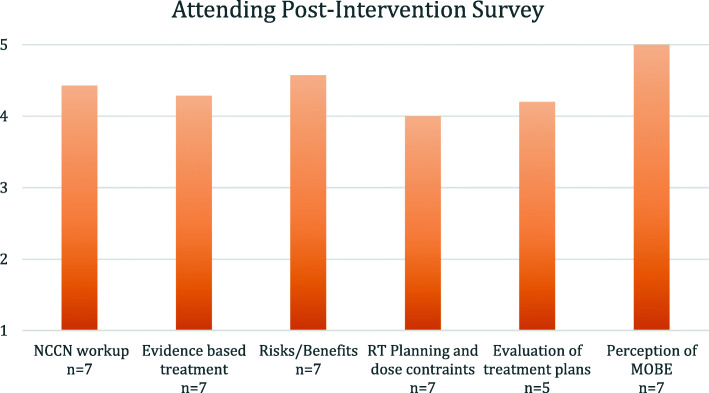
Attending post-intervention survey assessing global resident preparedness and personal perception of mock oral board examinations

All of the responding attendings (7/10 participating) characterized these MOBEs as “very beneficial,” and the average grade of all clinical skill themes was satisfied or above. The dose constraint and plan evaluation were numerically weaker [Fig. 4]. All 7 faculty members felt the overall resident clinical knowledge was appropriate for training level.

The qualitative resident feedback was universally positive with requests for more MOBE experiences moving forward. The most commonly identified barrier to program expansion was time commitment (for both examiner and examinee). Residents identified the following opportunities for improvement: standardizing the number of cases, greater inclusion of common disease site cases rather than “zebras,” and separating the feedback portion from the MOBE. These improvement suggestions were shared with faculty performing MOBE for future incorporation. Additionally, moving the program to earlier in the academic year would minimize overlap with PGY4 physics and radiation biology board exam studying.

## Discussion

This MOBE experience was well received by both residents and faculty. Encouragingly, there was a trend towards improved resident comfort with articulating their clinical skills following the MOBE experience. Barriers to future implementation were identified: time for preparation and implementation, and avoidance of conflict with other board preparatory endeavors. Of note, the residents and faculty did not identify conflict with clinical duties or other educational endeavors as a barrier, suggesting that implementation could occur without interruption of clinical services or educational programs already in place. Residents expressed a desire to modify future formatting and case composition to better emulate information likely to be tested on the OBE, and requested a temporally separate feedback session from faculty, which would serve to create a more realistic examination environment.

In addition to providing benefits to the trainees, the MOBE provides useful information to the program directors. MOBE have been shown to identify areas of clinical weakness, allowing targeted intervention to fill knowledge gaps [6, 7]. This MOBE experience provided similar results, with the identification of plan evaluation as an area in which our trainees felt less prepared. Based on our results, PGY4 residents appeared to feel less confident than PGY3s in some clinical areas, though this did not reach statistical significance. Given the PGY4s were in their elective year, it’s unsurprising their overall comfort with clinical skills is comparable to PGY3s as they had been out of clinic for at least 6 months focusing on research endeavors as well as studying for the physics and radiation biology board examination [11]. Alternatively, this may represent the natural progression of training, as earlier residents may feel like the volume of information is manageable, whereas later residents may understand the great degrees of nuance involved in medical decision-making.

The most robust experience supporting the use of MOBE comes from surgical education literature, which has correlated first-time American Board of Surgery (ABS) oral board pass rate with participation and success in MOBE [3, 4, 12, 13]. Additionally, these series demonstrate high levels of resident satisfaction following MOBE experience, [5] and found MOBE beneficial in preparing for the OBE, particularly due to exposure to common questions and test professionalism [4, 13]. Our current early experience is limited in that it does not have correlative data with trainee pass rates (no participating residents have taken the OBE to date), nor were faculty asked to assign a pass or fail grade. However, this is an area ripe for future study in radiation oncology. The educational utility of the MOBE is broad, including enhancement of the study process through focus on areas of perceived weaknesses, the discovery of areas of previously unidentified weaknesses, the improvement of verbal expression by the trainee, as well as the experiential learning that comes from having a rapid fire question and answer session with an attending. The MOBE experience allows for rehearsal of one’s verbal responses with adequate, but not superfluous detail. Often these discussions reveal a knowledge deficit, which can then result in high yield knowledge transfer between the MOBE participants. Additionally, the painful experience of sharing too much, and then being quizzed on the details of topics less deeply understood by the trainee, is valuable. The MOBE experience may also bolster confidence by demonstrating just how much mastery the trainee has already achieved. This rehearsal process itself, even without the additional learning, may decrease oral examination anxiety. We hypothesize these factors synergize to bolster resident knowledge and confidence in expressing their clinical skills for the oral exam.

This single institution pilot project may have limited applicability of its findings given the small number of participants. However, the median size of RO programs nationally is 8, [14] and therefore congruent with this sample. This program was developed due to a general sense among current trainees that MOBE should be a part of resident education, introducing inherent biases among the residents towards favorable perceived utility. However, the additional educational targets identified through this experience to program directors was not subject to these biases and suggests the utility is genuine. Another limitation of this work is the absence of formalized faculty training for MOBE administration. For our institution, this training was not felt to be necessary because of the 10 faculty participating in MOBE, 3 were current or previous oral board examiners and all remaining faculty had previously given ad hoc ABR mock oral examinations to recently graduated trainees. This familiarity with oral boards administration may be unique to our institution, and other institutions may need to consider a formalized training for faculty. It is striking that even without formalized training for faculty, this exercise was appreciated by trainees. This suggests that the forced practice of maintaining poise and professionalism during an oral exam may be even more important to trainee confidence than the content of the MOBE itself. Of note, the assessment of utility was made by trainees prior the experiencing the actual oral board examination. Therefore, they were not able to judge whose MOBE was more representative of the actual OBE when rating exercise benefit. The utility of MOBE at our institution may be strengthened by the adoption of formalized training to improve consistency of MOBE content.

## Conclusion

In conclusion, this educational pilot demonstrates the utility of MOBEs and suggests they are highly valued by both faculty and trainees, with unanimous agreement for increased future exposure to such experiences. Future directions for this MOBE program are inclusion of more disease sites, better emulation of the exam setting with the removal of immediate feedback, the creation of a more rigorous consolidated format testing all sites at once, and consideration for grading of these sessions for future correlation with OBE performance.

## Supplementary information

**Additional file 1.** Supplementary Index.

## Data Availability

All data generated or analyzed during this study are included in this published article.

## Abbreviations

RO: Radiation Oncology
ASTRO: American Society for Radiation Oncology
ABR: American Board of Radiology
OBE: Oral Board Exam
MOBE: Mock Oral Board Exam
PGY: Post-Graduate Year
QI: Quality Improvement
ABS: American Board of Surgery
