# Supplementary material for: Resident attitudes and benefits of mock oral board examinations in radiation oncology
Source: BMC Med Educ. 2020 Jun 26;20:203. doi: 10.1186/s12909-020-02106-4 (PMC7318518; doi:10.1186/s12909-020-02106-4)
Supplement: Supplementary file 1 — Additional file 1. Supplementary Index. [file 12909_2020_2106_MOESM1_ESM.docx]

Supplementary Index

1. Resident Pre-Intervention Survey

Resident Post-Intervention Survey

1. Mock Orals Resident Pre-Intervention Survey

- What year of post-graduate training are you currently in? (ie. PGY3, 4, 5)
- Have you ever participated in a structured one-on-one case based learning/socratic method experience for radiation oncology education?

Yes/No

- Were prior experiences valuable?

Yes/No

Likert Scale Questions

- On a scale of 1-5 how comfortable do you feel with the standard of care/NCCN guidelines for workup of malignancy?

1. Very Uncomfortable
2. Uncomfortable
3. Neutral
4. Comfortable
5. Very Comfortable

- On a scale of 1-5 how comfortable do you feel using evidence to support your treatment recommendation when speaking with attending physicians?

1. Very Uncomfortable
2. Uncomfortable
3. Neutral
4. Comfortable
5. Very Comfortable

- On a scale of 1-5 how comfortable do you feel weighing the risks/benefits of radiation treatment against the toxicity?

1. Very Uncomfortable
2. Uncomfortable
3. Neutral
4. Comfortable
5. Very Comfortable

- On a scale of 1-5 how comfortable do you feel formulating plan objectives and dose constraints for your dosimetrists?

1. Very Uncomfortable
2. Uncomfortable
3. Neutral
4. Comfortable
5. Very Comfortable

- On a scale of 1-5 how comfortable do you feel evaluating treatment plans?

1. Very Uncomfortable
2. Uncomfortable
3. Neutral
4. Comfortable
5. Very Comfortable

- On a scale of 1-5 how helpful do you find formal one-on-one, socratic method teaching?

1. Very Uncomfortable
2. Uncomfortable
3. Neutral
4. Comfortable
5. Very Comfortable

- How helpful do you find formal one-on-one, socratic method teaching?

1. Harmful
2. Not good
3. Neutral
4. Beneficial
5. Very Beneficial

- Based on response above, what makes socratic teaching beneficial/not beneficial?

2. Mock Orals Resident Post-Intervention Questions

- What year of post-graduate training are you currently in? (ie. PGY3, 4, 5)

Likert Scale Questions

- On a scale of 1-5 how comfortable do you feel with the standard of care/NCCN guidelines for workup of malignancy?

1. Very Uncomfortable
2. Uncomfortable
3. Neutral
4. Comfortable
5. Very Comfortable

- On a scale of 1-5 how comfortable do you feel using evidence to support your treatment recommendation when speaking with attending physicians?
  1. Very Uncomfortable
  2. Uncomfortable
  3. Neutral
  4. Comfortable
  5. Very Comfortable
- On a scale of 1-5 how comfortable do you feel weighing the risks/benefits of radiation treatment against the toxicity?

1. Very Uncomfortable
2. Uncomfortable
3. Neutral
4. Comfortable
5. Very Comfortable

- On a scale of 1-5 how comfortable do you feel formulating plan objectives and dose constraints for your dosimetrists?

1. Very Uncomfortable
2. Uncomfortable
3. Neutral
4. Comfortable
5. Very Comfortable

- On a scale of 1-5 how comfortable do you feel evaluating treatment plans?

1. Very Uncomfortable
2. Uncomfortable
3. Neutral
4. Comfortable
5. Very Comfortable

- On a scale of 1-5 how helpful do you find formal one-on-one, socratic method teaching?

1. Very Uncomfortable
2. Uncomfortable
3. Neutral
4. Comfortable
5. Very Comfortable

- How helpful do you find formal one-on-one, socratic method teaching?

1. Harmful
2. Not good
3. Neutral
4. Beneficial
5. Very Beneficial

- Do you feel the formal case-based learning and socratic method session has increased your clinical knowledge base and comfort in clinical reasoning?

Yes/No

- Do you feel more case-based learning experiences would be beneficial?

Yes/No

- What do you feel is the biggest barrier to having more of these sessions?
- How could these sessions have been better? Are there any additional comments you wish to provide?
